# Endogenous oxytocin response to film scenes of attachment and loss is pronounced in schizophrenia

**DOI:** 10.1093/scan/nsy110

**Published:** 2018-11-27

**Authors:** Lucas G Speck, Johanna Schöner, Felix Bermpohl, Andreas Heinz, Jürgen Gallinat, Tomislav Majić, Christiane Montag

**Affiliations:** 1 Charité—Universitätsmedizin Berlin, corporate member of Freie Universität Berlin, Humboldt-Universität zu Berlin; 2 Department of Psychiatry and Psychotherapy, Berlin Institute of Health, Campus Charité Mitte Charité Universitätsmedizin Berlin, Charitéplatz, Berlin, Germany; 3 Department of Psychiatry and Psychotherapy, University Medical Center Hamburg-Eppendorf, Hamburg, Germany

**Keywords:** schizophrenia, oxytocin, emotion induction, attachment, trauma

## Abstract

**Background:**

Oxytocin (OXT) is critically involved in the regulation of attachment and interpersonal function. In this study, emotional children’s movies were used to stimulate OXT secretion in patients with schizophrenia and healthy controls (HCs). Furthermore, associations of OXT levels with measures of attachment style (Psychosis Attachment Measure), childhood adversity (Childhood Trauma Questionnaire) and symptom severity [Positive and Negative Syndrome Scale (PANSS)] were considered.

**Methods:**

In 35 patients with schizophrenia and 35 matched HCs, radioimmunoassay with sample extraction was used to determine OXT plasma levels before and after viewing of movie scenes portraying emotional bonding and loss and compared to a non-emotional condition.

**Results:**

Statistical analysis indicated lower baseline OXT levels in female patients than in all other groups. OXT reactivity during emotional movies was significantly higher in patients when compared to HCs. OXT reactivity during the control movie related to PANSS `general psychopathology’. No significant associations appeared between baseline or induced OXT levels and other PANSS subscales, attachment style or childhood adversity in patients.

**Conclusions:**

Our findings suggest differences of baseline OXT and a higher OXT reactivity toward strong emotional stimuli in patients with schizophrenia, suggesting a role of OXT as a gender- and context-dependent modulator of socio-emotional function.

## Introduction

In recent decades, disturbances of empathy and emotional responsivity have gathered increasing attention in schizophrenia research. A reduced capacity to emotionally resonate with others, to understand and to regulate other induced feelings and relationship distress might complicate the formation of intimate bonds. In addition, individuals suffering from psychotic disorders are often burdened with substantial childhood adversity and may lack stable attachment experiences (Berry *et al.*, 2008). The spectrum of attachment-related dysfunctions, including conditions like autism, borderline personality disorder or schizophrenia, has been consistently linked to the oxytocinergic system. Oxytocin (OXT), a neurohypophyseal hormone and neurotransmitter, is considered to be an important modulator of social cognition and empathy (Domes *et al.*, 2007; Bartz *et al.*, 2011). Its prosocial effects have consistently been reported from studies investigating mother–infant (Feldman *et al.*, 2007) and pair bonding (Young and Wang, 2004), attachment (Buchheim *et al.*, 2009), cooperation (De Dreu and Kret, 2016) and trust (Kéri and Kiss, 2011). De Dreu and Kret (2016) speak of an OXT-biased, biobehavioral approach–avoidance principle, with OXT acting on social salience and reward, as well as dampening vegetative responses to social threats and stressors. Furthermore, OXT may facilitate affiliation by promoting in-group empathy, cooperation and trust, but also serve the protection from out-group danger by up-regulating vigilance and defense-motivated aggression. Therefore, OXT might affect more than one of the functional systems implicated in psychotic vulnerability by modulating attachment, social approach, interaction experience and mentalizing capacity on the one hand and the perception of aberrant salience and social threat on the other (Olff *et al.*, 2013; Debbané *et al.*, 2016). Early suggestions of OXT as a `natural antipsychotic’ and evidence from animal studies demonstrating OXT to counteract excessive mesolimbic dopamine and cortical hypoglutamatergia led to the assumption that OXT signaling might be altered in schizophrenia (Macdonald and Feifel, 2012). However, results from trials using intranasal OXT in patients with schizophrenia have been mixed, some reporting improvement of positive and negative symptoms or improvement of specific deficits, while others reported no benefit (Feifel *et al.*, 2016; Bradley and Woolley, 2017). Accordingly, the role of endogenous OXT remains inconclusive, with studies reporting lower (Goldman *et al.*, 2008; Jobst *et al.*, 2014; Aydin *et al.*, 2018) or higher (Legros *et al.*, 1992; Strauss *et al.*, 2015) cerebrospinal fluid (CSF) and plasma OXT levels in patients with schizophrenia, as well as varying associations with symptom load (Goldman *et al.*, 2008; Kéri *et al.*, 2009; Rubin *et al.*, 2010; Jobst *et al.*, 2014). A small number of studies report associations of plasma OXT levels with social cognitive capacity in schizophrenia patients (Goldman *et al.*, 2008; Walss-Bass *et al.*, 2013; Strauss *et al.*, 2015), the avoidance of angry faces (Brown *et al.*, 2014) or the perception of faces as happier (Rubin *et al.*, 2011). OXT measurements in the absence of a social stimulus seem highly variable, and therefore stimulus-evoked OXT level changes were considered to be more reliable (Zak *et al.*, 2005; Kéri and Kiss, 2011). In a single study, Kéri and coworkers showed OXT responses toward trust-related interactions to be blunted in schizophrenia patients (Kéri *et al.*, 2009).

The goal of this study was to compare baseline endogenous OXT levels as well as OXT level changes induced by social stimuli between groups of schizophrenia patients and healthy controls (HCs). To activate the endogenous OXT system, pivotal emotional scenes from children’s movies like `Bambi’ were shown to the participants. All selected scenes presented situations of bonding and loss of an attachment figure. The main hypotheses were that schizophrenia patients would (i) differ in OXT baseline levels when compared to HCs and (ii) show reduced OXT reactivity in the emotional, but not in a non-emotional control condition. On an exploratory basis, it was hypothesized that baseline and induced OXT levels in patients would be associated with (i) symptom severity, (ii) history of childhood trauma and (iii) attachment style, which have been linked with OXT dysfunction in previous studies (Heim *et al.*, 2009; Rilling, 2009).

## Materials and methods

### Participants

The study was approved by the local ethics committee; subjects gave written informed consent. Thirty-five in- and outpatients with paranoid schizophrenia (PS; 23 males), aged 18–65, were recruited from Charité Universitätsmedizin Berlin, Psychiatric University Clinic at St. Hedwig Hospital. Diagnosis and symptom severity were confirmed by the treating psychiatrist using structured clinical interviews for DSM-IV and the Positive and Negative Syndrome Scale (PANSS). All patients were stabilized, showing at best mild to moderate symptom load. PS showing antisocial personality traits were excluded (SCID I; SCID II items for antisocial personality disorder, German versions). Thirty-five HC subjects (HC; 23 males), matched for age and verbal IQ, were recruited by printed and direct verbal advertisement in the hospital and university (cleaning and nursing staff, students) and screened with structured interviews (SCID II, MINI; Table 1). Exclusion criteria for both groups were DSM-IV axis-I or axis-II disorders (except schizophrenia for patients), hormonal contraception, pregnancy and lactation. HC reporting axis-I mental disorders in their first- or second-degree relatives were excluded. Of note, none of the participants manifested postraumatic symptoms or PTSD. Participants had to abstain from alcohol/drug consumption 24 h prior to testing. Menstrual cycle phase of female subjects was based on the timeframe of their last menses. Coded as menses/follicular/luteal/menopause/unknown, distribution was *n* = 2/2/5/1/2 in the PS group, and *n* = 0/2/3/2/5 in HC women (χ^2^ = 4.119, *P* = 0.390).

**Table 1:** Demographic data and disease characteristics for schizophrenia patients and HCs

	HC	Schizophrenia	Statistics
Number of participants	35	35	
Gender (m/f)	23/12	23/12	^1^χ2 = 0.000
Age	36.0 ± 10.4	40.4 ± 8.8	^2^T = 1.882
Verbal IQ	114.1 ± 17.6	107.2 ± 18.0	^3^U = 471.000
AVLT^(1–5)^ score	10.7 ± 2.0	8.5 ± 2.2	^2^T = −4.340^***^
Educational years	14.2 ± 2.5	11.9 ± 2.4	^3^U = 140.000^**^
CTQ			
physical abuse	6.5 ± 3.6	7.2 ± 3.2	^3^U = 353.000
sexual abuse	5.2 ± 0.9	7.4 ± 4.4	^3^U = 295.000^***^
emotional abuse	7.7 ± 3.6	10.7 ± 4.1	^3^U = 238.000^***^
emotional neglect	9.5 ± 3.9	12.0 ± 4.6	^3^U = 318.000^*^
physical neglect	6.8 ± 2.3	8.7 ± 4.1	^3^U = 286.000^**^
PAM			
anxiety	2.1 ± 0.7	2.5 ± 0.5	^3^U = 278.000^***^
avoidance	2.0 ± 0.5	2.0 ± 0.4	^3^U = 507.500
Age at first manifestation	-	27.7 ± 8.9	-
Duration of illness	-	12.4 ± 8.4	-
Antipsychotic dose (CPZ, [mg])			
Total	-	386.4 ± 349.7	-
FGA	-	64.2 ± 148.6	-
SGA	-	304.2 ± 322.0	-
PANSS			
positive score^4^	-	18.2 ± 9.8	-
negative score^4^	-	19.7 ± 7.5	-
general psychopathology^4^	-	32.6 ± 11.0	-

^1^: χ2-Test; ^**2**^: T-Test for independent samples; ^**3**^: Mann–Whitney-U Test. ^*^: *P* < 0,05; ^**^: *P* < 0,01; ^***^: *P*< 0.001. Significant results are indicated in bold type. ^4^: *N* = 32. AVLT: Auditory Verbal Learning Test; CPZ: Chlorpromazine equivalent; CTQ: Childhood Trauma Questionnaire; FGA: first generation antipsychotics; PAM: Psychosis Attachment Measure; PANSS: Positive and Negative Syndrome Scale; SGA: second generation antipsychotics.

### Neurocognition

A multiple choice vocabulary test (Mehrfach-wahl-wortschatztest, MWT-B) was applied to estimate verbal intelligence. Additionally, a German version of the Auditory Verbal Learning Test (AVLT) was used as a measure of general cognitive functions such as multiple verbal memory components and executive functions. Means of the first five presentations (AVLT^(1–5)^) were used for analysis.

### Childhood adversity and attachment style

The 28-item form of the Childhood Trauma Questionnaire (CTQ-SF; Bernstein *et al.*, 2003) was used to assess adverse childhood experiences. This retrospective self-report measure assesses five types of maltreatment in separate subscales—emotional abuse, sexual abuse, physical abuse, emotional neglect and physical neglect. Each subscale includes five items that are rated on five-point Likert scales. Psychometric properties of the original five-factor solution were confirmed for the German translation (Dudeck *et al.*, 2015) and its usefulness was demonstrated in schizophrenia patients (Kim *et al.*, 2013). An authorized German translation of the 16-item Psychosis Attachment Measure (PAM; Berry *et al.*, 2008) estimated two aspects of insecure attachment, anxiety and avoidance (eight items each), by self-report, on a four-point Likert scale. Psychometric properties and factor structure of the English version are reported from a psychosis sample (Berry *et al.*, 2008).

### Video-stimulated OXT reactivity

For the induction of attachment-related feelings (emotion induction condition: EMOI), scenes from three children’s movies were chosen, giving an introduction to the relationship in question (bonding) and ending with the death of the attachment figure (loss, empathy with the main character). We chose the films `Bambi’ (6 min, 37 s), `The Lion King’ (5 min, 44 s) and `UP!’ (4 min, 21 s), portraying the loss of a mother, a father and a beloved wife, respectively. To obtain a reliable and strong emotion induction effect, all videos were presented consecutively in the same order and confronted participants with the same basic need of bonding and belonging, while romantic attachment played a minor role. A scene from a weather documentary represented the control condition (CON; 3 min, 37 s). The CON phase was shorter than the EMOI phase to prevent the induction of negative emotions like boredom. EMOI and CON films were balanced regarding their order of appearance (test version 1: EMOI first; test version 2: CON first). All subjects were tested between 10 a.m. and 1 p.m. to rule out natural fluctuations of OXT levels. Subjects were given a 60 min break between conditions.

After each movie scene, three questions were presented to the subject: (i) `How strongly did you feel with the main character?’ (EMPATHY), (ii) `How much have you felt stressed or fearful?’ (AROUSAL) and (iii) `How relevant is this scene to your life?’ (RELEVANCE). Subjects were asked to answer on a scale of 1 to 6. In the CON condition, the first question was replaced with (i) `How relaxed were you during the film?’ (RELAXATION), since the documentary did not feature any empathy-inducing character.

At debriefing after EMOI, participants were asked whether they would share their thoughts and feelings during the films with the experimenter. This was rated on a six-point scale ranging from `not at all’ to `completely’ (TRUST). Participants were then asked to take a short note of what they had felt.

A peripheral venous catheter was placed 30 min prior to testing. Before the first EMOI film and 1 min after the last EMOI film, as well as before and 1 min after the CON condition, citrated plasma samples were taken, centrifuged immediately and frozen (−28°C). The timing of plasma sampling was based on protocols using video-based stimulation or trust-related interventions (Barraza and Zak, 2009; Kéri *et al.*, 2009; Munro *et al.*, 2013).

OXT concentrations were determined by radioimmunoassay (RIA) using solid phase sample extraction by Prof. Dr Rainer Landgraf, RIAgnosis, Munich (http://www.riagnosis.com). Assay sensitivity for this method is in the 0.1 pg/ml sample range, intra- and inter-assay variability is under 10% and no significant cross-reactivity is reported. Details of extraction method, analysis and validation are reported elsewhere (http://www.riagnosis.com; Neumann *et al.*, 2013).

### Statistical analysis

Normal distribution was determined by Kolmogorov–Smirnov tests. OXT reactivity was calculated according the following equation:

$$ EMOI\ reactivity=\frac{\left[ Oxytocin\right]\  after\ EMOI\ films}{\left[ Oxytocin\right]\  before\ EMOI\ films} $$

$$ CON\  reactivity=\frac{\left[ Oxytocin\right]\  after\ control\ film}{\left[ Oxytocin\right]\  before\ control\ film}. $$

OXT reactivities were log-transformed to attain normal distribution [reactEMOI = log(EMOI reactivity); reactCON = log(CON reactivity].

## Results

Socio-demographic data, illness characteristics, CTQ-SF and PAM scores are shown in Table 1. PANSS measures for three patients were not available. Patients scored significantly lower than healthy subjects in AVLT^(1–5)^ values and educational years but indicated significantly more adverse childhood experiences on all CTQ-SF subscales apart from physical abuse, and more attachment anxiety on the PAM than controls.

### Behavioral data

PS showed significantly lower self-rated RELAXATION and higher AROUSAL values during the CON film than HC. No group differences appeared for EMPATHY, AROUSAL or subjective RELEVANCE related to the EMOI condition, but PS were slightly less willing to share their thoughts and feelings with the experimenter (TRUST; Table 2). No significant associations were detected between TRUST and PANSS total or subscores (all *P* > 0.05). Qualitative debriefing information was available from *n* = 31 PS and *n* = 28 HC. Topics differed between groups, while the emotional focus was reflected by the majority of participants in both groups (PS/HC: refused answer: 1/0; rational account: 4/1; emotional but impersonal account: 2/7; emotional personal recollection: 5/10; fear of loss and/or longing for attachment: 14/10; loneliness: 5/0).

**Table 2:** Self-ratings of emotional empathy, arousal or relaxation experienced during EMOI (sum) and CON, as well as personal relevance of stimuli, and willingness to trustfully share thoughts and feelings related to EMOI (TRUST), by patients with schizophrenia and HCs

	HCs	Schizophrenia	Statistical tests
EMOI films			
EMPATHY	13.63 ± 2.64	12.26 ± 3.50	U = 474.500
AROUSAL	6.14 ± 3.42	5.80 ± 2.49	U = 612.000
RELEVANCE	9.63 ± 4.09	8.63 ± 3.29	U = 536.500
TRUST	5.18 ± 1.19	4.56 ± 1.54	U = 392.500^*^
CON film			
RELAXATION	4.80 ± 1.05	4.07 ± 1.41	U = 428.500^*^
AROUSAL	1.20 ± 0.47	1.67 ± 1.13	U = 479.500^*^
RELEVANCE	2.48 ± 1.46	2.51 ± 1.65	U = 529.000

*N* = 35/35, means, s.d.; Mann–Whitney U test: *: *P* < 0.05; Significant results are indicated in bold type. EMOI: presentation of emotional films, CON: presentation of control film.

### Baseline OXT

Baseline OXT levels did not differ significantly between patients and HCs. Group comparisons of the male and female subsamples yielded no differences in males but showed significantly lower OXT levels in female patients compared to female controls (Table 3). Analysis of covariance (ANCOVA) was performed with log-transformed baseline OXT levels as the dependent variable, group and gender as factors and age as a covariate. There was a significant interaction between group and gender, F(1,70) = 5.663; *P* = 0.02, where female PS had lower baseline OXT levels than all other groups. There were no significant main effects of diagnosis, gender or age.

**Table 3:** Baseline OXT levels and reactivity of endogenous OXT (ratio OXT after/before film presentation) in schizophrenia patients and HCs

	HC	Schizophrenia	Statistics
OXT at baseline [pg/ml]	5.48 ± 4.50	4.59 ± 3.35	U = 576.000
Males	4.64 ± 3.84	5.38 ± 3.87	U = 218.000
Females	7.10 ± 5.36	3.07 ± 0.96	U = 31.000^*^
OXT EMOI reactivity	0.96 ± 0.38	1.22 ± 0.50	U = 371.000^*^
Males	1.01 ± 0.39	1.15 ± 0.42	U = 190.000
Females	0.85 ± 0.34	1.36 ± 0.64	U = 29.000^*^
OXT CON reactivity	1.07 ± 0.55	1.18 ± 0.48	U = 509.000
Males	1.08 ± 0.62	1.13 ± 0.49	U = 225.000
Females	1.04 ± 0.39	1.27 ± 0.47	U = 58.000

*N* = 35/35, means, s.d.; Mann–Whitney U test: *: *P* < 0.05; Significant results are indicated in bold type. EMOI: presentation of emotional films, CON: presentation of control film.

### OXT reactivity

PS showed significantly higher OXT levels after emotion/empathy induction (EMOI) than before (Wilcoxon test, Z = −2,129, *P* = 0.033), but in HC there were no differences before and after both conditions (EMOI: Z = −1,368, *P* = 0.171; CON: Z = −0,655, *P* = 0.512). Accordingly, PS showed a significantly higher OXT reactivity during emotion/empathy induction (EMOI) compared to HC (Table 3). To assess interaction effects between diagnostic group, gender and sequence of the experiments (version 1 *vs* version 2), a factorial multivariate analysis of variance (MANOVA) with logarithmized OXT reactivities (reactEMOI, reactCON) as dependent variables, and group, gender and test version as independent factors, followed by *post hoc* ANOVAs was run. Homogeneity of variances was confirmed by Box-M and Levene’s tests (*P* > 0.05). Partial eta^2^ (_p_η^2^*)* was used as an estimate of effect sizes. Significant main effects were found for group and test version (Table 4). *Post hoc* ANOVA revealed a significant effect of diagnostic group for reactEMOI, but not for reactCON, reactEMOI being significantly higher in PS, compared to HC. A significant effect of test version was found for the CON condition, but not for EMOI. ReactCON was significantly higher when CON was shown first. There were neither statistically significant interactions between group and gender or test version, nor between gender and test version, and no significant main effect of gender was found (Table 4).

**Table 4:** Group comparison of endogenous OXT’s reactivity (logarithmized ratio OXT after/before film presentation) in schizophrenia patients and HCs related to the presentation of emotional *vs* control films

	Group	Gender	Test version	Group X Gender	Group X Test version	Gender X Test version
MANOVA:						
F(2,62) *Effect size (_p_η^2^)*	4.657^*^ (0.131)	0.616 (0.019)	4.561^*^ (0.128)	0.874 (0.027)	1.593 (0.049)	1.024 (0.032)
*Post hoc* ANOVA: reactEMOI						
F(1,63) *Effect size (_p_η^2^)* reactCON	8.208^**^ (0.115)	0.701 (0.011)	0.663 (0.010)	1.328 (0.021)	2.029 (0.031)	0.214 (0.003)
F(1,63) *Effect size (_p_η^2^)*	2.803 (0.043)	0.312 (0.005)	7.344^**^ (0.104)	0.788 (0.012)	0.625 (0.010)	1.552 (0.024)

*N* = 35/35; MANOVA with logarithmically transformed OXT reactivities in the emotional films (reactEMOI) and in the control film (reactCON) as dependent variables; factors: group (HC/SZ), gender and test version (emotional films followed by control film/control film followed by emotional film). F[df], Effect size (_p_η^2^), ^*^: *P* < 0.05; ^**^: *P* < 0.01. Significant results are indicated in bold type.

Of note, the effect of diagnostic group on reactEMOI was not altered by inclusion of baseline OXT levels as a covariate (F[2,61] = 3.381, *P* = 0.041, _p_η^2^ = 0.10; *post hoc*: F[1,62] = 6.386, *P* = 0.014, _p_η^2^ = 0.14).

### Correlation analyses

On an exploratory basis, Spearman rank-order correlation coefficients served to assess the relationship between baseline OXT levels, OXT reactivities and socio-demographic and illness characteristics as well as measures of early adversity and attachment style.

Significant associations of baseline OXT, and OXT changes to EMOI or CON scenes with age, verbal IQ, AVLT^(1–5)^ scores or educational years could not be determined. There were no significant correlations of baseline OXT, EMOI or CON reactivity with any of the film behavioral questions (all *P* > 0.05).

A moderate negative correlation of baseline OXT and EMOI reactivity, but not CON reactivity, appeared in the PS group (r_s_ = −0.417, *P* = 0.013), but did not reach significance in HC (r_s_ = −0.304, *P* = 0.076), indicating that low OXT levels correlated with high OXT reactivity to the emotional films in persons with schizophrenia.

There was no significant association of PANSS subscales with baseline OXT levels or EMOI reactivity (all *P* > 0.05), but there was a moderate negative association of OXT CON reactivity with PANSS `general psychopathology’ scores (r_s_[32] = −0.460, *P* = 0.008). No significant associations were found between baseline OXT levels or reactivity and antipsychotic dose or duration of illness in PS or male and female PS subgroups (all *P* > 0.05).

No associations were found between baseline OXT, OXT reactivities and attachment measures (PAM anxiety and avoidance) or CTQ-SF total scores and subscales in both groups, even when analyzed separately by gender. When subjects with minimal *vs* low, moderate or severe childhood adversity (according to the cutoffs by Bernstein *et al.*, 2003) were compared, no significant differences in baseline OXT or OXT reactivity were observed in either group (all *P* > 0.05).

## Discussion

Results were partially consistent with our hypotheses: (i) baseline OXT levels were significantly lower in female patients than in healthy females; (ii) OXT reactivity during emotional films, but not during control films, was significantly higher in patients compared to controls and (iii) OXT reactivity during the control video, but not during emotional films, negatively related to PANSS `general psychopathology’ scores.

Our data suggest significantly lower baseline OXT levels in female patients than in healthy women, whereas no respective differences were found between male controls and male patients. Decreased baseline OXT levels have been reported in mixed samples of patients with schizophrenia with (Goldman *et al.*, 2008) or without (Aydin *et al.*, 2018) neuroendocrine dysfunction, and in males with schizophrenia (Jobst *et al.*, 2014), while other studies reported elevated OXT levels in male schizophrenia patients (Legros *et al.*, 1992) or in samples of more than 70% males (Walss-Bass *et al.*, 2013; Strauss *et al.*, 2015) compared to HCs. No group differences (Rubin *et al.*, 2010) and no significant group x gender interactions were reported, when medicated (Rubin *et al.*, 2014) or unmedicated (Rubin *et al.*, 2013) first episode patients were each compared to HCs. Studies analyzing CSF estimated higher (Beckmann *et al.*, 1985) or similar (Sasayama *et al.*, 2012) OXT levels in patients *vs* HCs. Current evidence regarding alterations of non-induced OXT levels in psychoses is not conclusive yet and difficult to interpret due to methodological issues; gender-specific differences were not regularly considered. Of note, comparing depressed individuals with HCs, reduced baseline OXT was found in females, but not in males (Ozsoy *et al.*, 2009), and results were suggested to reflect the complex interplay between OXT and gonadal hormones with a higher sensitivity of the female OXT system to the effects of stress (Ozsoy *et al.*, 2009).

In addition, a stronger influence of antipsychotic medication on OXT pathways in female patients could be discussed. Animal studies point to a direct influence of antipsychotic drugs on OXT pathways and release (Uvnäs-Moberg *et al.*, 1992; Kiss *et al.*, 2010), though human clinical evidence is still inconclusive (Beckmann *et al.*, 1985; Sasayama *et al.*, 2012). More likely, antipsychotics cause menstrual cycle irregularities (Murke *et al.*, 2011) and thus might indirectly suppress physiological OXT fluctuations by altering the estrogen-dependent regulation of the OXT-system (Gimpl and Fahrenholz, 2001). However, some studies did not find cycle-dependent variations of OXT levels in both female patients and healthy women (Rubin *et al.*, 2010, 2011), and there were no associations between OXT measures and antipsychotic dose in our study. Altogether, the topic of neuroleptic effects on the oxytocinergic system including possible interactions with gender remains to be further explored.

OXT reactivity during emotional films, but not during control films, was significantly higher in patients compared to controls. Again, this difference was most evident in females. To eliminate the possibility of the analysis having been skewed by differing baseline OXT levels between groups, we conducted a second analysis using baseline OXT as a covariate, which did not affect our results. To our knowledge, there is only one other study of induced peripheral OXT levels during social interaction in schizophrenia (Kéri *et al.*, 2009), reporting a blunted OXT response in patients compared to controls after sharing a secret with the experimenter. Compared to an experimental setting that probably leads to rather variable emotional responses, movie stimuli in the present study can be considered to reliably elicit strong attachment-related emotions, which was confirmed for both groups by personal debriefing. However, while female patients in our study showed increasing OXT levels during the experiment, a decrease was found in healthy women. This contrasts findings in healthy individuals showing OXT level increases in subjects viewing emotionally-laden movie scenes (Barraza and Zak, 2009). Interestingly, Munro *et al.*(2013) reported a modest rise of plasma OXT levels in females during a film’s bonding scene, but its significant decrease during an abandonment scene (Munro *et al.*, 2013). Our experimental setting did not allow for differentiation of bonding and abandonment effects, as each emotional video clip contained both a bonding and an abandonment phase. Therefore, personally salient aspects of the film may have differed between diagnostic groups and led to different physiological reactions, with patients more frequently reporting an unfulfilled desire for relationship or feelings of solitude at debriefing, stating that attachment figures like the ones shown in the films were what they `never had’. Healthy persons, who were more likely to have experienced close relationships, might have resonated more strongly with the fear of abandonment and loss. Alternatively, the `tend and befriend’ model (Taylor *et al.*, 2000) suggests an OXT release in response to stressors including social loss or threats thereof, thus increasing sensitivity and affiliative motivation (Gimpl and Fahrenholz, 2001; Onaka, 2004; Bartz *et al.*, 2011). This reaction type was found to be more prominent in women (Taylor *et al.*, 2000, 2010). As schizophrenia patients report higher personal distress when confronted with others in need, a higher susceptibility to contagion with negative emotions and reduced emotion regulation capacity (Lehmann *et al.*, 2014), film scenes of bonding and loss might cause an exaggerated experience of social stress and elicit `tend and befriend’ responses. Studies conducted in individuals suffering from depression or emotional distress suggest not only gender-specific reductions of baseline OXT (Ozsoy *et al.*, 2009), but also a dysregulated (Parker *et al.*, 2010) or more variable pulsatile OXT release (Cyranowski *et al.*, 2008). Higher OXT increases have been shown in response to relational stress (Tabak *et al.*, 2011) and in the course of an affiliation-focused imagery session, and might be related to interpersonal dysfunction (Cyranowski *et al.*, 2008). Of note, attachment-related stimuli might be more effective in eliciting OXT responses than general stress (Cyranowski *et al.*, 2008; Tabak *et al.*, 2011).

Patients’ increased vigilance toward social threat may additionally stimulate OXT release (De Dreu and Kret, 2016). Brown *et al.* (2014) demonstrated a stronger tendency to avoid angry faces, as well as more severe psychotic symptoms, in schizophrenia patients with higher baseline OXT levels. Accordingly, patients in our study may have been more susceptible to the induction of fear and distress, thus exhibiting a measurable difference in OXT release compared to controls. Consistent with previous studies in healthy subjects, this effect was more prominent in females (Barraza and Zak, 2009; Taylor *et al.*, 2010). The fact that self-ratings of perceived arousal and personal relevance of stimuli did not correspond to OXT increases during EMOI may be explained by the characteristic discrepancy between autonomous arousal and experiential aspects of emotion in schizophrenia (Kring and Neale, 1996).

Although no significant associations were observed between OXT measures and self-rated attachment styles in patients in this study, attachment anxiety was significantly more pronounced in patients than in controls. Insecure attachment styles have been associated with both higher stress responses and lower mean OXT levels compared to securely bound healthy subjects (Pierrehumbert *et al.*, 2012). OXT increases in response to attachment- or trust-related stimuli were found to be most prominent in individuals with insecure attachment representations (Kiss *et al.*, 2011; Krause *et al.*, 2016). In a similar vein, our patient group scored significantly higher than HCs in almost every CTQ subscale, but no associations with OXT levels and OXT reactivities could be determined, even if gender-specific aspects and the differential impact of different types of trauma were considered. Current evidence suggests associations between adverse childhood experiences and decreased OXT levels (Heim *et al.*, 2009; Opacka-Juffry and Mohiyeddini, 2012), possibly as a result of early programming differences in OXT neurocircuitry. Stress-induced OXT concentrations have been shown to be either higher in girls having experienced sexual abuse (Seltzer *et al.*, 2014), or lower in adults with childhood sexual abuse (Pierrehumbert *et al.*, 2010). Munro *et al.* (2013) found OXT increases induced by a film’s bonding scene in females with dissociative symptoms. However, we cannot exclude that differences in early childhood experience and attachment style may have modulated baseline and induced OXT levels in our patient sample, as very early interactional adversity during sensitive periods of development and disturbances of attunement and synchrony between child and caregiving persons might not be assessed by the CTQ (Feldman, 2015). Only two self-rating instruments and no objective reports were used to examine attachment representations and childhood adversity. Therefore, adverse circumstances like prematurity, maternal deprivation or maternal illness were not accounted for. Moreover, retrospective reporting might have been biased by paranoid symptoms in patients. Future research should focus on the relationship between early experience, attachment insecurity, OXT system dysregulation and psychotic vulnerability more deeply (Debbané *et al.*, 2016).

In contrast to schizophrenia patients, we observed decreased OXT levels after viewing emotional movie scenes in healthy women. Similarly, decreased OXT levels have been described as a response to negative emotional stimuli in healthy women (Turner *et al.*, 1999; Munro *et al.*, 2013). As we also observed significantly higher baseline OXT levels in female controls compared to patients, a physiological high activity of the oxytocinergic system might have attenuated reactions toward an emotional stressor. However, results regarding an association between peripheral OXT, distress or stress-related disorders are still not conclusive and `protective’ effects might be gender-specific and dependent on individual biographic factors (Taylor *et al.*, 2010; Weisman and Feldman, 2013; Olff *et al.*, 2013).

Another finding of our study was a significant main effect of test version on OXT reactivity, with higher OXT reactivity during the control stimulus, when it was presented first (test version 2). This could be due to several reasons. First, the expectation of an experimental manipulation, i.e. stress anticipation, might have impacted both experimental conditions at baseline and triggered stress-related OXT secretion (Olff *et al.*, 2013). As the EMOI stimuli on average caused higher arousal than the CON condition (Table 2), subtle stress anticipation effects might have become more obvious in the CON condition when presented first. Second, subjects in test version 1 had already watched the EMOI films, which might have lead to subsequent withdrawal of attention. Third, subjects watching the CON film first could not know that the later shown EMOI films were emotionally more impactful. Of note, watching a video about meteorological research, including diagrams and graphs, might be distressing, as indicated by significant differences in self-reports of patients and controls, and drive OXT release.

Our data do not suggest a link between baseline OXT levels and symptom severity. Previously, inverse relationships with peripheral OXT were reported for negative (Kéri *et al.*, 2009; Sasayama *et al.*, 2012; Jobst *et al.*, 2014), social, general (Rubin *et al.*, 2010) and social-cognitive symptoms (Goldman *et al.*, 2008; Strauss *et al.*, 2015), while findings regarding positive symptoms showed mixed results (Legros *et al.*, 1992; Rubin *et al.*, 2010, 2013; Brown *et al.*, 2014; Rubin *et al.*, 2014). In our patient group, a moderate negative correlation appeared between OXT reactivity and general psychopathology in the control condition, indicating that subjects with higher OXT reactivity to emotionally neutral scenes showed less general psychopathology. It can be speculated that less symptomatic patients were more reflective, performance-oriented and therefore more prone to stress anticipation effects. However, no associations were found for the EMOI condition. Future research might rather focus on the basic principles of OXT action in schizophrenia like attribution of social salience, motivation or anxiety in a more parsimonious experimental approach (Brown *et al.*, 2014).

### Limitations

Limitations of our study relate to the concern that peripheral OXT levels do not reliably reflect central processes. Although some evidence suggests that stress-related OXT responses can be confined to hypothalamic or limbic regions while having no immediate effect in peripheral OXT release, a recent meta-analysis indicates a positive correlation between peripheral and CSF OXT level changes when induced by stressors (Valstad *et al.*, 2017).

Moreover, no standard protocol exists for measuring OXT in blood samples. Comparability of research is hampered by the variety of (pre-)analytic procedures, but RIA with sample extraction is considered the most reliable and best-validated method (Szeto *et al.*, 2011; Neumann *et al.*, 2013; McCullough *et al.*, 2013). Though comparability of studies is very restricted, studies using video-based emotional stimulation and enzyme-linked immunosorbent assay (ELISA; Barraza and Zak, 2009; Kéri *et al.*, 2009; Munro *et al.*, 2013) reported results inconsistent with ours, while RIA-based evidence regarding induced OXT levels in conditions of emotional distress (Cyranowski *et al.*, 2008; Ozsoy *et al.*, 2009; Tabak *et al.*, 2011; Seltzer *et al.*, 2014) and in healthy persons (Turner *et al.*, 1999) might be less conflicting. However, research using RIA and extracted samples in patients with schizophrenia is limited to very few studies (Strauss *et al.*, 2015). Until OXT assaying in humans is not standardized against methods of references of high sensitivity and specificity, interpretation of results will remain unsatisfactory (McCullough *et al.*, 2013).

Another limitation of the study design refers to the fact that the time course of induced OXT release is not well known. No pilot testing of OXT peak levels was performed, but previous studies in healthy adults indicated measurable OXT increases immediately after 2 min of video-based emotional stimulation (Barraza and Zak, 2009) or after a short trust-related intervention (Kéri *et al.*, 2009). To obtain a reliable emotion induction effect on the one hand and to prevent the induction of negative emotions like boredom during the control condition on the other, EMOI and CON phases were of different duration, and OXT increases during the emotional condition could therefore be not specific. In addition, it might have required more frequent measurements in timed intervals across emotion induction and recovery phases to detect group differences regarding the specific temporal dynamics of induced OXT release.

Unfortunately, neither estradiol nor prolactin as potential mediators of OXT release and their complex interactions were a focus of this study.

Further limitations refer to a possible influence of social interaction with the experimenter on OXT reactivity (Kéri *et al.*, 2009), which was not systematically evaluated, the heterogeneity of the samples regarding menstrual cycle phase and the lack of control for sexual activity.

## Conclusion

This study corroborates previous evidence of an alteration of the oxytocinergic system in schizophrenia by measuring peripheral OXT at baseline and after stimulation in an attachment-related experimental setting. A deeper knowledge of endogenous OXT regulation, considering its individual and biographic context and interactions with antipsychotic and mood-stabilizing agents, may forward a more personalized use of intranasal OXT as a pharmacological treatment strategy.
